# The bacterial Mrp_ORP_ is a novel Mrp/NBP35 protein involved in iron-sulfur biogenesis

**DOI:** 10.1038/s41598-018-37021-8

**Published:** 2019-01-24

**Authors:** Romain Pardoux, Anouchka Fiévet, Cíntia Carreira, Céline Brochier-Armanet, Odile Valette, Zorah Dermoun, Béatrice Py, Alain Dolla, Sofia R. Pauleta, Corinne Aubert

**Affiliations:** 1 Aix Marseille Univ, CNRS, LCB, Marseille, France; 2 Microbial Stress Lab. UCIBIO, REQUIMTE, Department Química, Faculdade de Ciências e Tecnologica, Universidade NOVA de Lisboa, Campus da Caparica, Caparica, 2829-516 Portugal; 3 Univ Lyon, Université Lyon 1, CNRS, UMR5558, Laboratoire de Biométrie et Biologie Évolutive, 43 bd du 11 novembre 1918, F-69622 Villeurbanne, France; 4 Aix Marseille Univ, Université de Toulon, CNRS, IRD, MIO, Marseille, France

## Abstract

Despite recent advances in understanding the biogenesis of iron-sulfur (Fe-S) proteins, most studies focused on aerobic bacteria as model organisms. Accordingly, multiple players have been proposed to participate in the Fe-S delivery step to apo-target proteins, but critical gaps exist in the knowledge of Fe-S proteins biogenesis in anaerobic organisms. Mrp/NBP35 ATP-binding proteins are a subclass of the soluble P-loop containing nucleoside triphosphate hydrolase superfamily (P-loop NTPase) known to bind and transfer Fe-S clusters *in vitro*. Here, we report investigations of a novel atypical two-domain Mrp/NBP35 ATP-binding protein named Mrp_ORP_ associating a P-loop NTPase domain with a dinitrogenase iron-molybdenum cofactor biosynthesis domain (Di-Nase). Characterization of full length Mrp_ORP_, as well as of its two domains, showed that both domains bind Fe-S clusters. We provide *in vitro* evidence that the P-loop NTPase domain of the Mrp_ORP_ can efficiently transfer its Fe-S cluster to apo-target proteins of the ORange Protein (ORP) complex, suggesting that this novel protein is involved in the maturation of these Fe-S proteins. Last, we showed for the first time, by fluorescence microscopy imaging a polar localization of a Mrp/NBP35 protein.

## Introduction

Iron-Sulfur (Fe-S) metalloproteins are key players of a wide range of important biological processes, such as gene expression, DNA repair, RNA modification, central metabolism and respiration. *In vivo*, the biogenesis of Fe-S cluster requires complex multiproteic machineries. In prokaryotes, three Fe-S cluster biogenesis pathways have been identified: ISC, SUF and NIF^1–4^. Each of these three pathways requires three main steps: i) the acquisition of iron atom from an undiscovered donor and sulfur atom from cysteine by a cysteine desulfurase, ii) the assembly of the Fe-S cluster on a scaffold protein and iii) the Fe-S cluster delivery to apo-target proteins either directly from the scaffold protein or through carrier proteins that subsequently traffic and transfer the Fe-S cluster to the apo-target proteins.

In eukaryotes, Fe-S clusters are also synthesized by dedicated biogenesis pathways located in the plastids, mitochondria and cytosol. Plastids contain the SUF pathway while mitochondria use the ISC pathway. Cytosolic and nuclear Fe-S proteins, assisted by the ISC system, are maturated by a set of nine cytosolic components known as the CIA pathway for Cytosolic Iron-sulfur protein Assembly^3,5,6^.

The first component of the CIA machinery to be identified was the essential and highly conserved Cfd1, isolated from yeast^7^. This protein belongs to the Mrp/NBP35 ATP-binding protein subclass (also called ApbC) of P-loop containing nucleoside triphosphate hydrolase found ubiquitously in the three domains of Life^8^. Members of this subfamily, studied so far, have been shown to play a role in Fe-S biogenesis. For instance, Nbp35 and Cfd1 proteins form an heteromeric scaffold complex in yeast and mammalian CIA pathway, whereas mitochondrial Ind1, belonging also to the Mrp/NBP35 ATP-binding protein subclass, serves as Fe-S cluster carrier to mature respiratory Complex I in yeast^9^. Also, in bacteria, the *Salmonella enterica* ApbC protein has been shown to be required for the maturation of TcuB, an enzyme necessary to use tricarbalyllate as carbon source and it was proposed to exhert a Fe-S cluster carrier role^10,11^.

Mrp/NBP35 ATP-binding proteins harbor two main signatures: a deviant Walker A motif (GKGGhGK[ST]) involved in ATP binding and a conserved CXXC motif^12–14^. The ATPase activity has been demonstrated *in vitro* for the *Salmonella enterica* ApbC and Yeast Nbp35 proteins but the role of nucleotide hydrolysis in cluster biogenesis still remains unclear^15–17^. The conserved CXXC motif is involved in the binding of a bridging [4Fe-4S] cluster between monomers and is essential for the Fe-S biogenesis *in vivo*^15,16,18^.

Curiously, few bacterial proteins belonging to the Mrp/NBP35 ATP-binding subclass have been studied to date whereas they are ubiquitous in microorganisms. Orp9 (DVU2109) is a protein composed of two domains with one of them showing homologies with Mrp/NBP35 proteins. Orp9 is a component of the ORangeProtein (ORP) complex from the Sulfate Reducing Microorganism (SRM) model organism, *Desulfovibrio vulgaris* Hildenborough (*Dv*H)^19^. The ORP complex is made of 5 proteins named Orp3 (DVU2103), Orp4 (DVU2104), Orp5 (DVU2105), Orp8 (DVU2108) and Orp9 (DVU2109), putatively involved in cell division^19^. The genes coding for ORP proteins are clustered into two divergent operons whose expression is controled from a σ^54^-dependent promoter^19,20^. Most of the ORP proteins are predicted to be metal binding proteins. Hence, Orp3 and Orp4 share 41% similarity to each other and each protein contains two conserved [4Fe-4S] ferredoxin binding motifs (IPR017900), Orp8 is homologous to proteins harboring a non-covalently metal sulfide [S_2_MoS_2_CuS_2_MoS_2_] cluster^21,22^ and Orp5 is homologous to a protein that binds [2Fe-2S] clusters^23^. The actors involved in the maturation of the ORP complex metallo-proteins are yet unknown.

In this work, we show that Orp9 from *Dv*H and its homolog Dde3202 from *Desulfovibrio alaskensis* G20 (*Dd*G20) contain a Mrp/NBP35 domain and we report the biophysical and biochemical characterization of both proteins. We demonstrate that these two proteins possess two domains, each able to bind a Fe-S cluster. We also evidence that only the Mrp/Nbp35 domain can efficiently transfer its Fe-S cluster to apo-targets, such as aconitase and Orp proteins. We propose that Orp9 and its homolog Dde3202, named hereafter Mrp_ORP_, constitute a novel type of Mrp/Nbp35-like proteins required for the maturation of the Fe-S cluster containing proteins of the ORP complex. Finally, we provide the first localization of a Mrp/NBP35 protein in a microorganism.

## Results

### The Mrp_ORP_ proteins are two-domain proteins

Querying the INTERPRO portail indicated that Mrp_ORP_ belongs to the Mrp/NBP35 ATP-binding proteins (IPR019591), a very large protein family ubiquitous in the domains of Life (Supplementary Table S1). This protein family encompasses the prokaryotic Mrp and ApbC, and the eukaryotic Nbp35 and Cfd1 proteins involved in Fe-S cluster biogenesis^12,16,24^. Most of the 17,511 members of the Mrp/NBP35 ATP-binding protein family contained a single conserved functional domain, the P-loop containing nucleoside triphosphate hydrolase domain (P-loop NTPase, IPR027417). However, in a few cases this domain is found associated with other domains (Supplementary Table S2). In Mrp_ORP_, the P-loop NTPase domain is associated with a dinitrogenase iron-molybdenum cofactor biosynthesis domain (Di-Nase, IPR003731), which is usualy found in proteins involved in the biosynthesis of the iron-molybdenum cofactor (FeMo-co), such as NifB and NafY^25^. The association between P-loop NTPase and Di-Nase domains was observed in 99 members of the Mrp/Nbp35 ATP-binding family (Supplementary Table S3). They corresponded mainly to proteins from anaerobic organisms, such as *Thermodesulfobacteria*, *Clostridia* and *Desulfovibrio* (Supplementary Table S3).

**Figure 1:**
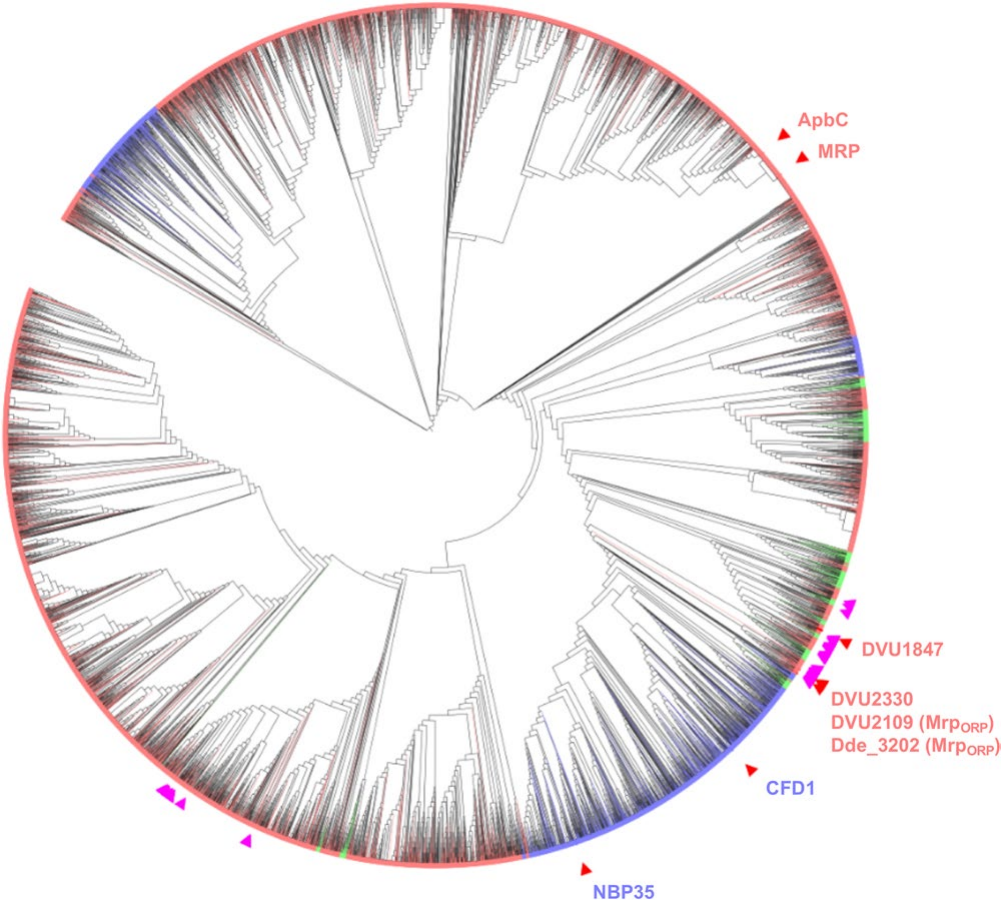
Unrooted Maximum likelihood tree of the Mrp/Nbp35 ATP-binding protein family (IPR019591, 16154 sequences, 114 amino acid positions conserved for the analysis after trimming). The tree is displayed as a cladogramme. Branch colors correspond to taxonomic groups: pink = *Bacteria*, blue = *Eucarya*, green = *Archaea*. DVU2109, DVU1847, and DVU2330 from *Desulfovibrio vulgaris* strain Hildenborough ATCC 29579, Dde3202 from *Desulfovibrio alaskensis* strain G20, Mrp from *Escherichia coli* strain K12, ApbC from *Salmonella typhimurium* strain LT2 SGSC1412 ATCC 700720, Cfd1 and Nbp35 from *Saccharomyces cerevisiae* strain ATCC 204508 S288c are indicated by red triangles. The 99 sequences associating a P-loop NTPase domain and a Di-Nase domain are indicated by purple triangles. Because of the large number of sequences contained in the tree, some triangles may overlap.

The phylogenetic analysis of the Mrp/NBP35 ATP-binding protein family showed that sequences from the three domains of Life are mixed on the tree (Fig. 1), indicating that horizontal gene transfers among domains occurred during the diversification of this protein family. According to this phylogeny, the Mrp_ORP_ protein is more related to the eukaryotic Nbp35 and Cfd1 than to the bacterial ApbC and Mrp (Fig. 1, purple triangles). The 99 sequences harboring an association between the P-loop NTPase and Di-Nase domains do not form a monophyletic group (Fig. 1, pink triangles). In fact, they belong to different parts of the tree, indicating that the association between these two domains occurred several times independently. The phylogenetic analysis of the 110 sequences displaying the highest sequence similarity with the P-loop NTPase domain of Mrp_ORP_ disclosed its closest relatives that are mainly from *Deltaproteobacteria* and revaled again phylogenetic relationships at odd with the current systematics confirming that the evolution of the Mrp/NBP35 ATP-binding family has been heavily impacted by horizontal gene transfers (Supplementary Fig. S1). Again, sequences harboring both the P-loop NTPase and Di-Nase domains appeared intermixed with sequences containing only the P-loop NTPase domain, confirming that such an association occurred mainy times during evolution.

**Figure 2:**
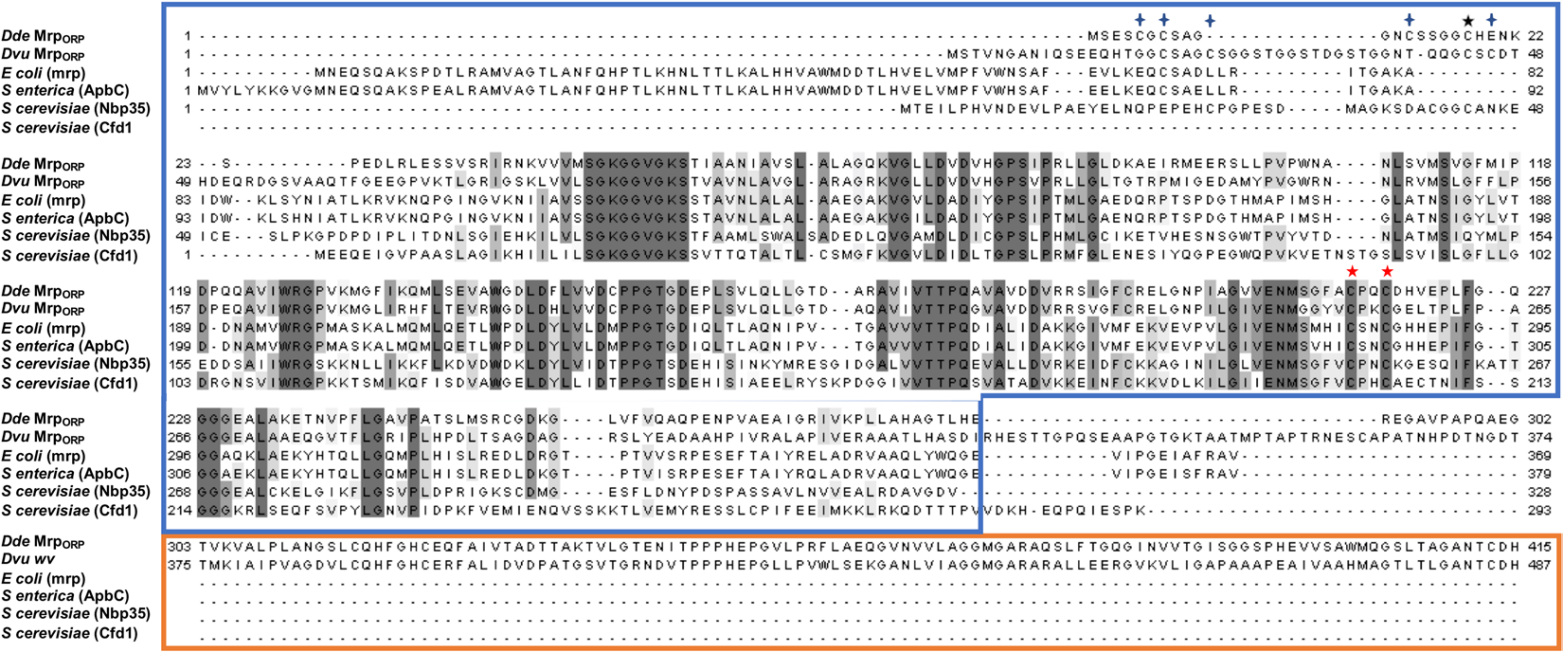
Protein sequences alignment of bacterial Mrp/ApbC and eukaryotic Nbp35 homologs. Bacterial sequences are from Dde (DDE_3202, UniProt accession no. Q30WF0), *Dv*H (DVU2109, UniProt accession no. Q72A88), *E*. *coli* (Mrp, UniProt accession no. P0AF08) and *S*. *enterica* (ApbC, UniProt accession no. Q8ZNN5). The eukaryotic homologs are the paralogs from *S*. *cerevisiae* (Nbp35, UniProt accession no. P52920 and Cfd1; UniProt accession no. P40558). Conserved amino acids are shown on a black background. Conserved cysteine residues are indicated with an asterisk black or a blue cross above the residue. Mutated cysteine in alanine residues are indicated with a red asterisk above the residue. The blue frame corresponds to the P-loop NTPase end the orange to the Di-Nase domain. The sequence alignment was built using the clustal O program https://www.ebi.ac.uk/Tools/msa/clustalo/ (version 1.2.1).

Genes coding for Mrp_ORP_ proteins from the sulfate reducer deltaproteobacteria *Dv*H and *Dd*G20 were both located in the *orp* genes cluster (Supplementary Fig. S2)^19,22^. The Mrp_ORP_ proteins from *Dv*H and *Dd*G20 have a molecular mass of 50 kDa and 43 kDa, respectively, with a P-loop NTPase domain of 30 kDa and a Di-Nase domain of 13 kDa for Mrp_ORP_ with DvH exhibiting a suplementary linker between the two domains (Fig. 2). The sequence alignment of the two Mrp_ORP_ with *E*. *coli* Mrp, *S*. *enterica* ApbC, *S*. *cerevisiae* Nbp35 and Cfd1, showed that the typical deviant Mrp Walker A (GKGGhGK[ST]), Walker B motifs, and CXXC motifs are conserved in Mrp_ORP_ (Fig. 2)^12,16,26^. We found also that in the Mrp_ORP_, four cysteine residues were present in the N-terminal part of the P-loop NTPase domain of Mrp_ORP_ proteins (Fig. 2, blue crosses) with only one of them conserved in eukaryotic Nbp35 (Fig. 2, black asterisk). These cysteine residues might form a non-canonical motif: CX_3_CX_20_CXC in Mrp_ORP_ of *Dv*H and CXCX_5_CX_4_C in Mrp_ORP_ of *Dd*G20.

Yet, the association between a P-loop NTPase and a Di-Nase domains raises the question about the role of Mrp_ORP_ proteins. We then investigated the biochemical properties of this new type of Mrp-like protein.

### The conserved CXXC motif from the P-loop NTPase domain of Mrp_ORP_ binds a Fe-S cluster

**Figure 3:**
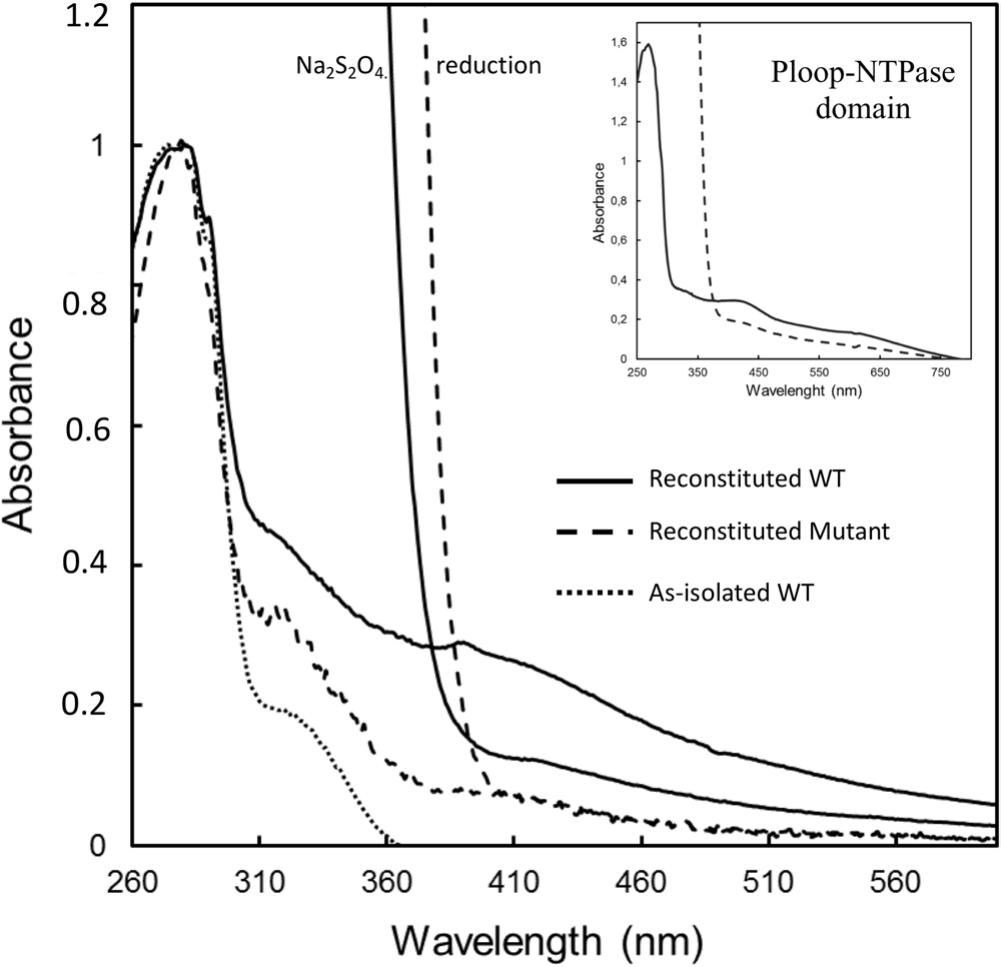
UV-visible absorption spectra of oxidized and reduced wild-type full, P-loop NTPase domain and mutant Mrp_ORP C215A-C218A_ proteins. UV-visible absorption spectra of aerobically isolated wild-type Mrp_ORP_ (dotted line)_,_ enzymatically reconstituted the Mrp_ORP_ protein before (solid bold line) and after reduction with addition of sodium dithionite (solid grey line) and enzymatically reconstituted Mrp_ORPC215A,C218A_ protein before (dashed bold line) and after reduction with addition of sodium dithionite (dashed grey line). Inset: UV-visible absorption spectra of the enzymatically Fe-S reconstituted P-loop NTPase domain before (solid line) and after reduction with addition of sodium dithionite (*dashed line*).

We, first, investigated the presence of a Fe-S cluster bound to Mrp_ORP_. The UV-visible spectrum of the aerobically isolated *Dd*G20 Mrp_ORP_ purified from *E*. *coli*, showed no absorbance corresponding to a Fe-S signature (Fig. 3, dotted line). However, after *in vitro* Fe-S cluster reconstitution under DTT reducing conditions in the presence of iron (Fe^2+^) and sulfur (S^2−^ or cysteine), the Mrp_ORP_ domain proteins exhibited a broad peak at 400 nm with a shoulder at 325 nm typical of 4Fe-4S clusters (Fig. 3, black solid line). The A_400_/A_280_ ratio of the reconstituted protein is 0.3. Upon reduction with dithionite, the absorbance at 400 nm decreased in comparison with the oxidized spectra (Fig. 3, grey solid line). Iron quantification showed that the full-length Mrp_ORP_ contained 5.25 ± 0.05 Fe per polypeptide chain. In the same way, Fe-S cluster reconstitution of the P-loop NTPase domain led to 4Fe-4S clusters signature but with a A_400_/A_280_ ratio of 0.18 lower than the full-length protein (Fig. 3, Inset). The two conserved cysteine residues, Cys215 and Cys218, of the Mrp_ORP_ CXXC motif were then replaced by alanine residues by site directed mutagenesis (Fig. 2, red asterisks). The UV-visible absorption spectrum of the corresponding reconstituted variant protein Mrp_ORP_C215A/C218A exhibited a drastic decrease in the absorbance at 400 nm with an A_400_/A_280_ ratio of 0.08 compared to 0.3 for the wild-type protein (Fig. 3, dashed line).

From these results, we conclude that the CXXC motif of the P-loop NTPase domain of Mrp_ORP_ is involved in the binding of a Fe-S cluster.

### The Di-Nase domain of Mrp_ORP_ binds a 3Fe-4S cluster

**Figure 4:**
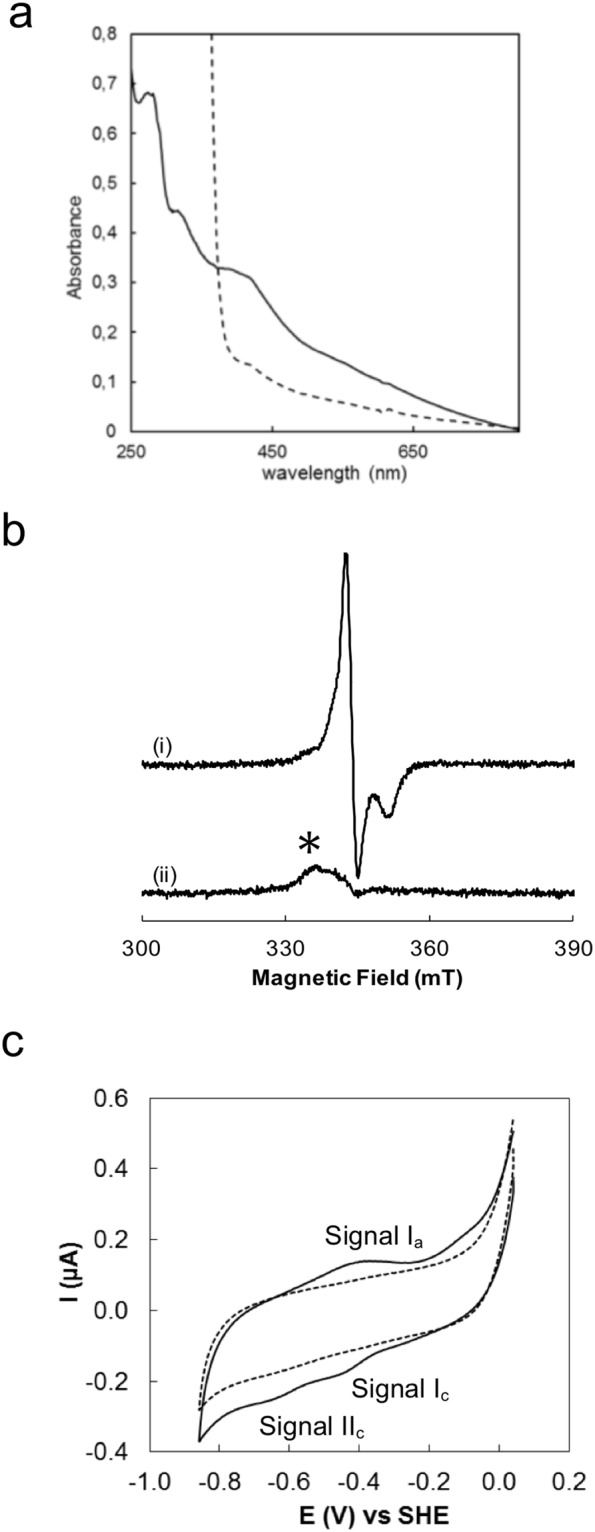
The Di-Nase of Mrp_ORP_ binds a 3Fe4S cluster. (**a**) UV-visible absorption spectra of the anaerobically purified Di-Nase domain of Mrp_ORP_ protein isolated from *Dv*H before (solid line) and after reduction by addition of sodium dithionite (dashed line). (**b**) EPR spectra of 80 μM (i) as prepared and (ii) dithionite reduced Mrp_ORP_ in 50 mM Tris-HCl 8.1, 150 mM NaCl, 1 mM DTT. Instrument settings: microwave frequency, 9.66 GHz; modulation amplitude, 5 G; microwave power, 6 mW; gain, 1 × 10^5^ temperature, 20 K. An impurity signal, designated by * arises from a trace species present in the cavity baseline. (**c**) Cyclic voltammogram of 121 µM Di-Nase domain of Mrp_ORP_ on a PGE electrode coated with 1.5 µL of polymyxin B sulphate (2 mM), in 100 mM Tris-HCl pH 8.0, 500 mM NaCl, 3 mM DTT and 2.5 mM desthiobiotin at a scan rate of 20 mV.s^−1^. Dash line represents the blank voltammogram and solid line the voltammogram of the protein film. The arrow indicates the direction of the scan.

We noticed that the reconstituted Mrp_ORP_C215A/C218A exhibited a weak absorbance around 400 nm compared to the apo-protein spectrum and that the P-loop NTPase domain had a ratio A_400_/A_280_ lower than the full-length Mrp_ORP_ (Fig. 3). To explore the hypothesis that the Di-Nase domain could bind a Fe-S cluster, the strep-tagged-Di-Nase domain of Mrp_ORP_ (Mrp_ORP__CT) was anaerobically produced and isolated from *Dv*H. The as-isolated domain has a brown color and exhibited an UV-visible spectrum with a broad absorption band at 420 nm, with a shoulder at 325 nm (Fig. 4A, solid line), and a A_420_/A_277_ ratio equal to 0.57. These features disappeared upon reduction with sodium dithionite (Fig. 4A, dashed line). The Mrp_ORP__CT contained 3.2 ± 0.1 Fe per polypeptide chain, and an extinction coefficient of 15200 M^−1^ cm^−1^ at 420 nm (with an extinction coefficient per iron of around 4750 M^−1^ cm^−1^), a value within the expected range for Fe-S cluster-containing proteins. As previous studies described Mrp/Npb35 proteins (Nbp35, Ind1 and ApbC) as dimeric proteins^9,12,16,26^, the quaternary structure of Mrp_ORP__CT was analyzed by gel filtration (Supplementary Fig. S3). The Mrp_ORP__CT domain eluted mainly as a dimer (Supplementary Fig. S3).

The Mrp_ORP__CT exhibits an EPR signal in the as-isolated form, in the perpendicular-mode, with g values at 2.012, 2.009 and 1.96, that decreases in intensity by increasing the temperature (data not shown), while the dithionite-reduced form is EPR silent (Fig. 4B) in this measurement mode. These EPR spectral features and metal quantification point to the presence of a [3Fe-4S] center, in the [3Fe-4S]^1+^ oxidation state. Considering that there is a [3Fe-4S]^1+^, this center presents three high-spin ferric ions, which are spin coupled, with a S_total_ = 1/2 state, and upon reduction to the [3Fe-4S]° oxidation state, by one electron, yields an integer spin (S_total_ = 2) species, which is not observed in the perpendicular measurement mode (Fig. 4B). The g-values are not similar to the ones found in typical [3Fe-4S] cluster containing proteins, but this protein does not present in its primary sequence the usual binding motif for this type of metal cluster. Nevertheless, the g-values determined for Mrp_ORP__CT are close to the ones reported for ThiI that has been shown to bind a [3Fe-4S] cluster^27^.

Therefore, the spectroscopic data together with the presence of only 3 conserved cysteine residues in the primary sequence of this domain (Fig. 2), support the hypothesis of a cuboidal [3Fe-4S]^1+^ cluster (*S*_total_ = 1/2) being present in Mrp_ORP__CT, that can be reduced to [3Fe-4S]° (S_total_ = 2)^28^.

The cyclic voltammogram of Mrp_ORP__CT presents a reversible signal at −445 ± 10 mV (signal I in Fig. 4C) and another at −645 ± 10 mV (signal II in Fig. 4C) of which it is only observed the cathodic counterpart. The first reduction potential corresponds to the pair [3Fe-4S]^1+^/[3Fe-4S]°, while the lower reduction potential is attributed to the reduction of [3Fe-4S]^0^ to [3Fe-4S]^−1^, which is not reversible in the conditions tested.

In conclusion, the spectroscopic and redox properties of Mrp_ORP__CT are consistent with the presence of a [3Fe-4S] cluster in the Di_Nase domain.

### Mrp_ORP_ transfers its Fe-S cluster to apo-aconitase

**Figure 5:**
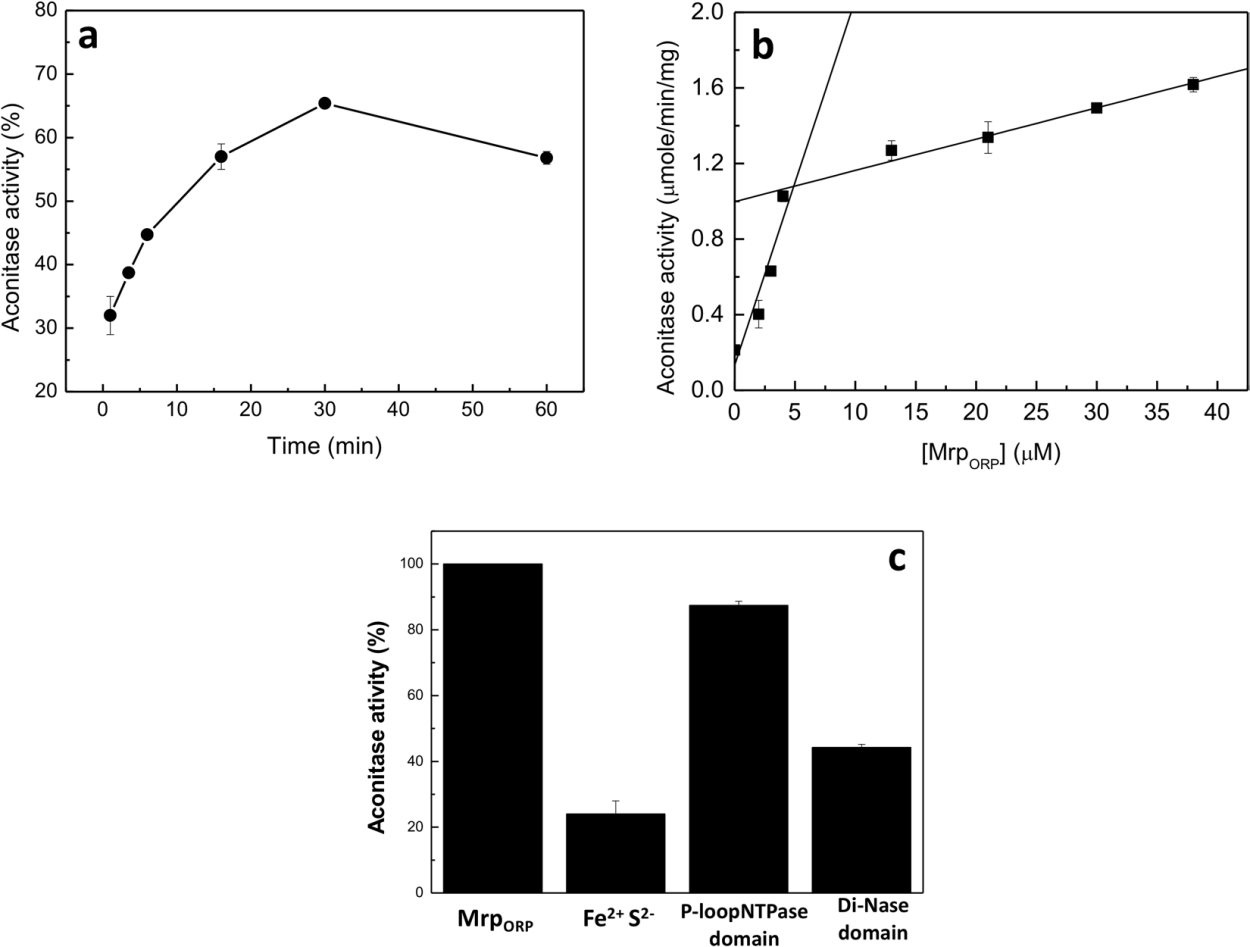
Reconstituted Mrp_ORP_ is able to transfer its [Fe-S] clusters to Apo-AcnB. The graph shows the correlation between aconitase activity and pre-incubation time between Rec-Mrp_ORP_ (16 µM) and Apo-AcnB (2 µM). 100% of activity corresponds to the specific activity of AcnB recorded with reconstituted AcnB in the same condition. Data points show the average of two experiments. (**b**) Correlation between the increasing level of Rec-Mrp_ORP_ and aconitase activity (full square). AcnB activation assays contained 2 µM of apo-AcnB protein and 0 to 40 µM of Rec-Mrp_ORP_. Apo-AcnB and reconstituted Mrp_ORP_ proteins were incubated together during 30 minutes before recording aconitase activity. Aconitase activity was measured at 30 °C during 1 h at 340 nm under anaerobiosis. (**c**) Aconitase activities relative to the full-length Rec-Mrp_ORP_ determined with 15 µM of Fe^2+^ and S^2−^Rec-, 15 µM of P-loop NTPase domain, 20 µM of Di-Nase domain and 4 µM of Rec_AcnB. Data are represented as the average of two independent experiments with standard deviations shown as error bars.

We then analyzed whether holo-Mrp_ORP_ was able to transfer, *in vitro*, Fe-S clusters to apo-protein, such as Aconitase B (AcnB). AcnB is known to be active only when its [4Fe-4S] cluster is inserted into the protein^29^. AcnB was purified aerobically from recombinant *E*. *coli* and Fe-S cluster was completely removed to obtain an inactive apo-AcnB. Reconstituted Mrp_ORP_ was then incubated with Apo-AcnB in an anaerobic chamber in the presence of DTT and the enzymatic activity of AcnB was determined at periodic time intervals. The AcnB activity increased with time in the presence of a fixed concentration of holo-Mrp_ORP_ (Fig. 5A). No significant AcnB activities were observed when apo-Mrp_ORP_ was used instead of holo-Mrp_ORP_ (data not shown) or when 15 μM of Fe^2+^ and S^2−^ were added instead of holo-Mrp_ORP_ (Fig. 5C).

Using a fixed concentration of apo-AcnB with various concentration of holo-Mrp_ORP,_ we determined the amount of holo-Mrp_ORP_ necessary to activate apo-AcnB (Fig. 5B, full square). Approximately 4 μM of holo-Mrp_ORP_ were required to activate 2 μM of AcnB, *i*.*e*. in a ratio of 2 molecules of holo-Mrp_ORP_ for 1 of AcnB (Fig. 5B, full square). As the presence of about 4 moles of iron and sulfide atoms are necessary to aconitase to be active^29^, a homodimer of holo-Mrp_ORP_ sharing 4 iron and 4 sulfide atoms are transferred to apo-aconitase. The holo-Mrp_ORP_ is then able to transfer its Fe-S cluster to apo-aconitase.

Additionally, we found that the reconstituted P-loop NTPase domain of Mrp_ORP_ transferred its Fe-S cluster to apo-AcnB resulting in an AcnB activity comparable to the full-length protein (87.4%) (Fig. 5C). When the same experiment was performed with the holo-Di-Nase domain of Mrp_ORP_ an aconitase activity of 44% of the full-length Mrp_ORP_ activity was observed (Fig. 5C).

Altogether, these results show that, *in vitro*, the Fe-S cluster that is transferred from Mrp_ORP_ to AcnB is preferentially the one bound to the P-loop NTPase domain.

### Mrp_ORP_ is able to transfer its Fe-S clusters to its physiological ORP partners

**Figure 6:**
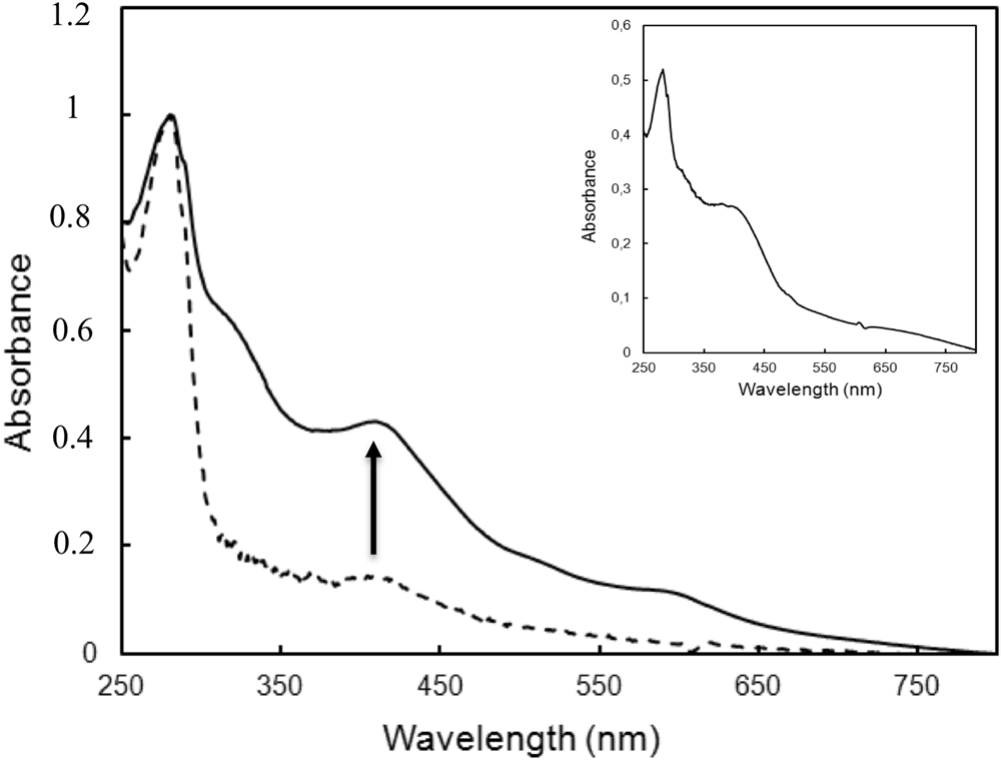
Mrp_ORP_ transfers its [Fe-S] cluster to ORP protein_S_. Apo-Orp3-Orp4-Orp8 proteins (dashed line) were mixed with Holo-rp_ORP_ during 90 minutes in anaerobic condition. After separation on a Ni-NTA column, the UV-visible absorption spectrum of the eluate fraction containing Orp3 was recorded (solid line). Inset: UV-visible spectrum of holo- Orp3-Orp4-Orp8 purified in anaerobiosis condition from *Dv*H.

We previously showed that Mrp_ORP_ interacts *in vivo* with the ORP complex that contains Fe-S binding proteins, especially the Orp3 and Orp4 proteins that each exhibits two [4Fe-4S] ferredoxin-like motifs^19^. We thus tested whether Mrp_ORP_ was able to transfer its Fe-S clusters to its physiological partner proteins. For this purpose, from *Dv*H cell extract, the His-tagged Orp3, that we systematically co-purified with its partners Orp4 and Orp8, was treated to remove metal centers (Fig. 6, dashed line). *In vitro* Fe-S transfer assays were performed using the reconstituted holo-form of a Strep-tagged Mrp_ORP_ and the His-tagged apo-Orp3-Orp4-Orp8 complex. The reconstituted holo-Mrp_ORP_ and apo-Orp3-Orp4-Orp8 were incubated for 90 min under anaerobic conditions and the proteins were separated using a Ni-NTA column. After separation, the UV-visible spectrum of the eluted fraction exhibited strong absorption bands at 420 nm and 325 nm with an A_400_/A_280_ ratio of 0.42, a spectrum similar to the spectrum of anaerobically purified holo-Orp3-Orp4-Orp8 proteins exhibiting A_400_/A_280_ ratio of 0.53 (Fig. 6, solid line and inset). These results demonstrate that, *in vitro*, Mrp_ORP_ can efficiently transfer Fe-S cluster to its physiological partner, the ORP complex.

### Mrp_ORP_ exhibits a polar localization in *Dv*H

**Figure 7:**
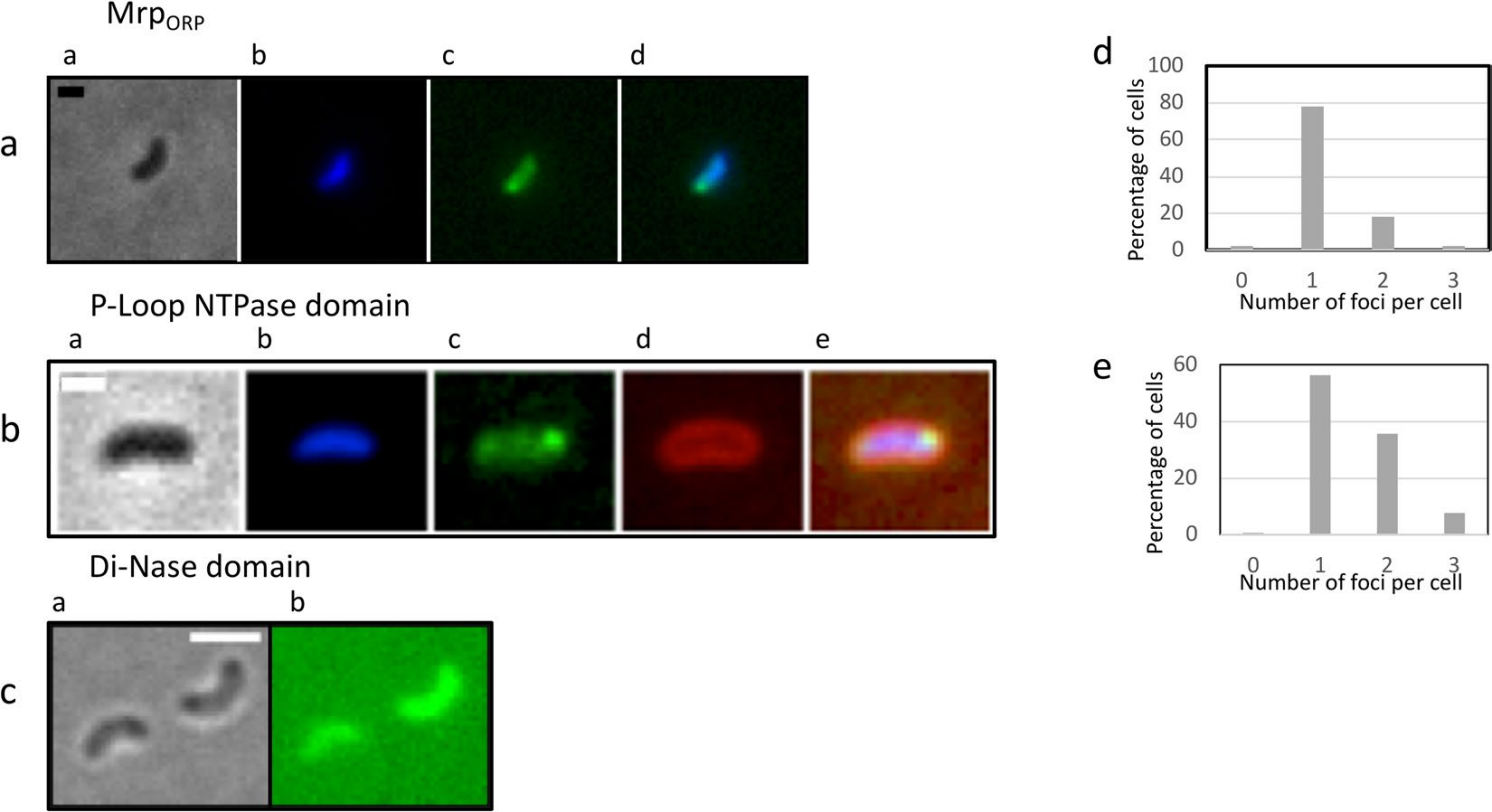
Polar and dynamic subcellular localization of Mrp_ORP_-GFP. Subcellular localization of Mrp_ORP_-GFP. (**a**) Subcellular localization of full length Mrp_ORP_-GFP. The first column represents the phase-contrast images (a), the second represents the nucleoid localization (DAPI) (b), the third represents the Mrp_ORP_-GFP fluorescence (c) and the fourth represents an overlay of both fluorescence signals (d). Scale bar = 1 µm. (**b**) Subcellular localization of the P-loop NTPase-GFP fusion protein. The first column represents the phase-contrast images (a), the second represents the nucleoid localization (DAPI) (b), the third represents the P-loop NTPase -GFP fluorescence (c), the fourth represents the membrane localization (FM4–64) (d) and the sixth represents an overlay of all flurescence signals (e). Scale bar = 1 µm. (**c**) Subcellular localization of the Di-Nase-GFP fusion protein. The first column represents the phase-contrast images (a) and the second represents the Mrp_ORP_-GFP fluorescence (b). Scale bar = 2 µm. (**d**) Statistical analysis of the the number of foci per cell located at the cellular pole in Mrp_ORP_ during the initiation step of the cell cycle of *Dv*H was performed using 362 cells from 3 independent experiments. (**e**) Statistical analysis of the number of foci per cell located at the cellular pole of the P-Loop NTPase domain during the initiation step of the cell cycle of *Dv*H was performed using 1232 cells from 3 independent experiments.

To further characterize Mrp_ORP,_ we then assessed its cellular localization in *DvH* using fluorescence microscopy imaging (Fig. 7). To achieve this goal, the full length Mrp_ORP_ was fused to the green fluorescent protein (GFP). In order to express the fusion *mrp*_*ORP*_*-gfp* gene from the native promoter, the fusion was introduced into the *Dv*H chromosome at the *orp* locus, replacing the endogenous wild-type *mrp*_*ORP*_ gene. Because GFP does not fluoresce in the absence of oxygen, cells were first grown under anaerobic conditions, and the pictures were acquired less than 10 min after contact with air. From our previous study, we showed that this time of air incubation doesn’t affect the localization of a FtsZ-GFP fusion in *Dv*H^30^. The profile of the fluorescence signal observed during the initiation step of the cell cycle of *Dv*H for Mrp_ORP_ appeared to be localized at one pole for 78% of the cells (Fig. 7A). Western blot analysis of the Mrp_ORP_-GFP using anti-GFP antibodies revealed only one band corresponding to the fusion protein, Mrp_ORP_-GFP, suggesting that the integrity of the fusion proteins was conserved (Supplementary Fig. S4). The P-loop NTPase domain was mostly located at one (58% of cells) or two poles (37% of cells) (Fig. 7B) whereas the fluorescence of the GFP-Di-Nase fusion was diffused in the cytoplasm (Fig. 7C). No growth defect was observed whatever the recombinant strain used.

These results revealed a polar spatial localization of Mrp_ORP_ linked to the Mrp/NBP35 domain.

## Discussion

P-loop NTPases are one of the largest class of proteins with subgroup members involved in a wide variety of essential cellular functions^8^. The Mrp/NBP35 ATP-binding protein subclass comprises proteins present in all three kingdoms of life and mainly involved in Fe-S cluster biogenesis^7–11,15^.

In this study, we characterised Mrp_ORP_, a novel type of Mrp/NBP35 ATP-binding protein. Mrp_ORP_ is distinct form the other members of this family by the fact that it associates a P-loop containing nucleoside triphosphate hydrolase domain (P-loop NTPase) with a dinitrogenase iron-molybdenum cofactor biosynthesis domain (Di-Nase). The phylogenetic analysis of the Mrp/NBP35 ATP-binding protein family showed that the association between both domains occurred several times independently.

Characterization of the reconstituted wild-type and mutant Mrp_ORP_ proteins, with the results described from biochemical analyses of other members of the Mrp/NBP35 ATP-binding protein family, indicated that the conserved CXXC motif of the P-loop NTPase domain coordinates a [4Fe-4S] cluster between two Mrp_ORP_ molecules. Interestingly, our data suggest that, in spite of the lack of classical Fe-S binding motif, the Di-Nase domain does have a 3Fe-4S cluster that can exist in two redox states [3Fe-4S]^1+^ and [3Fe-4S]^0^, with a reduction potential of −445 mV. The hypothesis that the observed [3Fe-4S] center could correspond to the degradation of a [4Fe-4S] is put aside by the fact that similar data were obtained from two different preparations and all the manipulation were performed inside the anaerobic box in a one-day purification procedure. However additional work is needed for definitive identification of this cluster. From our preliminary results, the cluster in the Di-Nase domain of Mrp_ORP_ seems to be clearly different from the heterometalic sulfide cluster (S_2_MoS_2_CuS_2_MoS_2_) noncovalently bond to the polypeptide chain of Orp8, another Di-Nase one-domain protein belonging to the ORP complex^31,32^. It is also different from the IssA protein from *Pyrococcus furiosus* belonging to the same family and that binds thioferrate through a cationic sequence in the C-terminal tail not found in Orp9^33^. A multiple alignment of the Di-Nase domain of proteins associating this domain with the P-loop NTPase domain as in Mrp_ORP,_ shows two cysteine_histidine rich conserved motifs: the CXHFGHCE motif located at the beginning of the Di-Nase domain and the CDH sequence located at the end of the domain (Supplementary Fig. S5). As cysteine and histidine residues can coordinate a Fe-S cluster, our hypothesis is that these conserved residues are involved in the binding of the [3Fe-4S] cluster in the Di-Nase domains of Mrp_ORP_.

Altogether, these results allow us to propose that Mrp_ORP_ is a novel member of the Mrp/NBP35 ATP-binding family which can bind at least two Fe-S clusters, one interdomain [4Fe-4S] cluster in the P-loop NTPase domain and one [3Fe-4S] cluster in the Di-Nase domain.

We further showed that the Fe-S cluster of Mrp_ORP_ can be transferred to apo-proteins, such as the aconitase and the ferredoxin-like proteins (Orp3 and Orp4) of the ORP complex previously shown to interact with Mrp_ORP_ *in vivo*^19^. The genomic clustering of *mrp*_*ORP*_ with the ORP encoding genes, further, is in total agreement with the idea that Mrp_ORP_ might be dedicated to the maturation of the Fe-S containing metalloproteins belonging to the ORP complex. To date, the only known target identified for bacterial Mrp is the TcuB protein, a protein necessary for tricarballylate catabolism^10^. We propose here the ORP complex as a novel target of Mrp/NBP35 ATP-binding proteins.

We then investigated the cellular localization of Mrp_ORP_ that was mainly observed at one pole of the cell. Such polar localization for a Fe-S carrier has never been described before and questions the localization of others prokaryotic Mrp/NBP35 ATP-binding protein because we showed that the polar localization is probably linked to the P-loop NTPase domain. Interestingly, this localization is found to be in adequation with the putative apo-target localization, as Orp3 and Orp4 exhibit a C-terminal amphipatic helix shown in MinD to be responsible for polar binding (Fig. S6)^34^. Polar localization was previously observed for protein involved in several biological processes, such as cell division, chemotaxis, signal transduction, cellular differentiation, virulence and bacterial respiration^35–40^ but never reported for a Fe-S protein maturation factor.

We demonstrated that the Fe-S cluster present in the Di-Nase domain is not efficienly transferable to apo-aconitase. Then, the role of this domain is still unclear. The presence in the Di-Nase domain of a high content of conserved proline residues (TPPPHXPGXXP), that have been shown to be involved in protein-protein/domain interaction, might be responsible for the specificity of interaction of Mrp_ORP_ with dedicated apo-partners to which the Fe-S is transferred (in magenta in Supplementary Fig. S5)^41^. Alternatively, the Fe-S cluster present in the Di-Nase domain might have a structural role in Mrp_ORP_. Hence, the presence of these unusual [3Fe-4S] clusters has been observed in enzymes, such as nitrate reductase, [NiFe] hydrogenase and ThiI^27,42,43^. Although, their role in those proteins has not been established, those centers have been considered to be involved in electron transfer and recently in sulfur transfer as in Thil^27,43^.

It has been shown that Nbp35 proteins possess an extra stable 4Fe-4S cluster absent in others Mrp/NBP35 ATP-binding proteins characterized to date^13,24^. The role of this 4Fe-4S located in the N-terminal extension of Nbp35 is still unclear. Curiously, Mrp_ORP_ contains also four cysteine residues included in a non-canonical motif in the N-terminal part of the P-loop NTPase with only one cysteine residue conserved in Nbp35 (Fig. 2). This feature added to the phylogenic position of Mrp_ORP_, suggest that bacterial Mrp_ORP_ are closer to eukaryotic Nbp35 than bacterial Mrp and ApbC.

Fe-S cluster biogenesis in anaerobic bacteria is poorly documented, while these organisms rely heavily on Fe-S cluster enzymes^44^. This study starts to fill this gap by describing that Mrp_ORP_ is likely an Fe-S cluster biogenesis factor in *Dv*H and *Dd*G20. Genome scanning analysis revealed that *Dv*H possesses a minimal ISC system constituted by a cysteine desulfurase and a scaffold protein (NifU type)^45^. In addition, we detected homologues of the *E*. *coli* SufB and SufD proteins that might constitute a minimal SUF system, reminiscent of what is observed in archaea. To date, Mrp/NBP35 ATP-binding proteins in Fe-S biogenesis have been proposed to act as Fe-S scaffold and carriers^12,16,24^. Interrestingly, we noticed redundancy of Mrp proteins in *Dv*H and other SRM. In *Dv*H, two other Mrp/NBP35 ATP-binding proteins are detected, DVU1847 and DVU2330, and are composed solely of the P-loop NTPase domain containing the conserved deviant Walker box and the CXXC motif. *Dvu2330* belonged to an operon encoding proteins involved in the biogenesis of Fe-S hydrogenases and *Dvu1847* is included in an operon encoding a L-isoaspartate O-methyltransferase. The outstanding redundancy of Mrp/NBP35 ATP-binding proteins in *Dv*H and other SRM raises the question of the role of each of these proteins in these anaerobic microorganisms. Our phylogenomic study shows clearly that the three Mrp/Nbp35 ATP-binding proteins from *Dv*H are closer to the eukaryotic Mrp/NBP35 than the bacterial and archaeal proteins.

Future studies will determine whether the assembly of Fe-S cluster on Mrp_ORP_ is dependent of the general Fe-S biogenesis of *Dv*H (ISC or SUF) or if Mrp_ORP_ acts in parallel of these systems.

## Methods

### Bacterial Strains, Plasmids and Growth Conditions

Strains and plasmids used in this study are listed in Table S4. *Escherichia coli* DH5α and TG1 strains were grown in Luria-Bertani (LB) medium at 37 °C with the appropriate antibiotic when required (0.27 mM for ampicillin, 0.15 mM for chloramphenicol). Cultures of *Dv*H were grown in medium C^46^ at 33 °C in an anaerobic atmosphere supplemented with 0.17 mM of kanamycin or 0.15 mM of thiamphenicol when required. Anaerobic work was performed using an anaerobic chamber (COY Laboratory Products or MBraun) filled with a 10% H_2_-90% N_2_ mixed-gas atmosphere. Before placement inside the anaerobic chamber, solutions were made anoxic by flushing with N_2_ for removal of O_2_. Solutions, glass and plastic materials were equilibrated for at least 12 hours inside the anaerobic chamber before use.

### Construction of Plasmids Used for Protein Production in *E*. *coli*

Standard protocols were used for cloning, ligation and transformations. Custom oligonucleotides used are listed in Table S5. All restriction endonucleases and DNA modifications enzymes were purchased from New England Biolabs. Plasmids DNA were purified using the High Pure Isolation Plasmid Kit (Roche Diagnostics). PCR products were purified using MiniElute kits (Qiagen). For construction of pJF119-2109His, pJF119-3202His and pJF119-Nter3202His the appropriated primers described in Table S5 were used to amplify the *dvu2109* and *dde3202* genes from genomic DNA. The obtained PCR products and the pJF119 plasmid were digested with *EcoR*I and *BamH*I restriction enzymes and ligated into the multiple cloning site of the plasmid to obtain pJF119-2109His, pJF119-3202His and pJF119-Nter3202His, respectively. For all constructs, successful ligations were confirmed via DNA sequencing and subsequently transformed into TG1 *E*. *coli* cells.

### Construction of Plasmids Used for Protein Production in *Dv*H

For construction of pBMC6C3::3202strep, pBMC6C3::Cter2109strep and pBMC6C3::2103His the appropriated primers described in Table S5 were used to amplify the *dde3202*, *dvu2109* and *dvu2103* genes from genomic DNA. The obtained PCR products and the pBMC6C3 plasmid were digested with *Nde*I and *Sac*I restriction enzymes and ligated into the multiple cloning site of the plasmid to obtain pBMC6C3::3202strep, pBMC6C3::Cter2109strep and pBMC6C3::2103His, respectively. For all constructs, successful ligations were confirmed via DNA sequencing and subsequently electroporated into *Dv*H cells.

### Site Directed Mutagenesis

Simultaneous mutations of cys215 and cys218 residues from Mrp_ORP_ were generated by oligonucleotide-directed mutagenesis using pBMC6C3::3202His as the PCR template and the Q5 site directed mutagenesis kit from Biolabs. The primers 3202cysmutF and 3202cysmutR were designed using the online NEB primer design software NEBaseChanger^TM^.

### Construction of the DvH (Mrp_ORP_-GFP) Strains

In order to produce a fusion Mrp_ORP_-GFP, Ploop-NTPase-GFP and Di-Nase-GFP in DvH cells, a non-replicative plasmid with the fusions *mrp*_*ORP*_*-gfp*, *ploop-ntpase-gfp and di-nase-gfp* were constructed and inserted into the *mrp*_*ORP*_ locus. With this construction, although the wild-type copy of *mrp*_*ORP*_ is still present, it does not present the σ^54^ binding site unlike the *Mrp*_*ORP*_*-GFP* allowing the expression of the fusion of interest in physiological conditions. The *mrp*_ORP_ amplicon obtained by using the primer pair NterDVU2109_*Xho*I/CterDVU2109_*Nde*I and the plasmid pNot19Cm-Mob-XS*-gfp*^30^ were cut with *Xho*I and *Nde*I. A gel extraction of the plasmid pNot19Cm-Mob-XS-*gfp* was done to insert *mrp*_*ORP*_ into this plasmid using the *Xho*I and *Nde*I sites to obtain the plasmid pNot19Cm-Mob-XS-*mrp*_ORP_-gfp. To obtain, pNot19Cm-Mob-XS *ploop-ntpase-gfp* and pNot19Cm-Mob-XS-*di-nase-gfp*, the amplicons *ploop-ntpase-gfp* and *di-nase-gfp* were amplified by PCR using pNot19Cm-Mob-XS *mrp*_*orp*_*-gfp* as template and the primer pairs Nter2109-*Xho*I/CterGFP-*Spe*I and domCter2109-dir-*Xho*I/CterGFP-*Spe*I, respectively. The amplicons were cut with *Xho*I and *Spe*I and inserted into the plasmid pNot19Cm-Mob-XS. The 3 plasmids were then transferred into *E*. *coli* WM3064 and subsequently transferred by conjugation to *Dv*H cells. Cells carrying the chromosomal recombination with the target fusion were selected for their resistance to thiamphenicol and checked by PCR using primers pair: DVU2108_UP/CterGFP-*Spe*I. A western blotting on *Dv*H cells was also down by using an anti-GFP antibody, as described by Fievet *et al*., 2015 to control the production of Mrp_ORP_-GFP fusion^30^.

### Protein Production and Purification

Proteins were obtained as follows: 1 mM isopropyl β-d-thiogalactoside (IPTG) was added to an exponentially growing culture at 37 °C of recombinant *E*. *coli* strains in 2 L of LB medium containing 100 µg/mL ampicillin and grown for additional 4 h at 37 °C. *Desulfovibrio* strains were cultivated in 20 L of medium C until the optical density at 600 nm reached 0.8 to 0.9. Proteins were purified aerobically in a cold refrigerated cabinet or anaerobically in an anaerobic chamber with identical purification protocols. For Mrp_ORP_ and Orp3-Histidine tagged proteins, the bacterial pellet was resuspended in buffer A (100 mM Tris-HCl, pH 7.5, 500 mM NaCl, 3 mM DTT) containing 30 mM imidazole, DNase I (Roche) DNAse and cOmplete^TM^ protease inhibitor EDTA free (from Roche Applied Science). Cell suspensions were disrupted two times in a chilled French pressure cell (4 °C) French press (Thermo-FA-080A) at 1200 psi. Cell lysates were clarified by centrifugation (45000 g for 80 min at 4 °C). After filtration of the supernatant across a 0.2 µm filter, the soluble proteins were loaded onto a 5 mL Ni-Sepharose affinity column (His Trap HP, GE Healthcare) equilibrated with buffer A with 30 mM imidazole. Mrp_ORP_ poly-histidine tagged proteins were eluted with buffer A containing 300 mM imidazole. Orp3- poly-Histidine tagged protein was eluted with buffer A containing 100 mM imidazole. The same procedure was applied to purify Mrp_ORP_ Strep tagged protein except that the soluble fraction was loaded onto a 5 mL Strep Trap affinity column (Strep Trap^TM^ HP, GE Healthcare) equilibrated with buffer A. Orp3 Strep tagged protein was eluted with buffer A supplemented with 2.5 mM desthiobiotin. Mrp_ORP_CT was purified inside an anaerobic chamber. The cells were resuspended in 100 mM Tris-HCl pH 8.1, 500 mM NaCl and 3 mM DTT buffer containing DNAse I (Roche) and protease inhibitors (EDTA-free cOmplete^TM^ Protease Inhibitor, Roche). The suspension was disrupted with a French press (Thermo-FA-080A) at 1200 psi and then flushed with argon during 30 min, to ensure a more anoxic environment, followed by centrifugation (Beckman Avanti J-26 XPI) at 6000 g for 30 min at 4 °C. The supernatant was degassed again for 30 min and then centrifuged at 45000 g, for 1 h at 6 °C. The soluble extract was flushed with argon and loaded onto a StrepTrap HP column (GE Healthcare) equilibrated with 100 mM Tris-HCl pH 8.1, 500 mM NaCl and 3 mM DTT. Mrp_ORP__CT was eluted with 2.5 mM desthiobiotin. When necessary, if the protein is not pure enough, a second purification step was performed using a Superdex 75 10/300 (GE Healthcare) equilibrated with 50 mM Tris-HCl, pH 7.6, 150 mM NaCl, 1 mM DTT. Protein purity was analyzed in a 12.5% Tris-Tricine SDS-PAGE. The fraction containing the protein was concentrated with centrifugal filter units (cut-off of 5 kDa) and freezed in liquid nitrogen until further use. Aconitase (AcnB) was purified as described for AcnA^47^. After purification of recombinant proteins, the eluted fractions were buffer exchanged with the buffer specified using a Hitrap Desalting column (GE Healthcare). Fractions that contained protein of interest at >95% purity, by SDS-PAGE analysis, were pooled and concentrated over a 10 kDa molecular mass cutoff membrane. Finally, the proteins were stored in liquid nitrogen. Protein concentration was determined using a Pierce^TM^ 660 nm Protein Assay (Thermo) Pierce colorimetric assay. Bovine serum albumin (2 mg/mL, Sigma) was used as a standard.

### [Fe-S] Cluster Reconstitution

[Fe-S] cluster reconstitution was performed anaerobically in an anaerobic chamber (COY) at 18 °C as follows. Protein was reduced anaerobically with 5 mM DTT for at least 1 hour prior to Fe^2+^ and S^2−^ addition. After pre-reduction, FeCl_3_ was added to five-fold excess and incubated for approximately 2 minutes before an addition of a 5-fold excess of Li_2_S. The solution was incubated for 4 hours before excess salts and unbound iron were removed using a Hitrap Desalting column (GE Healthcare). For enzymatic reconstitutions, 5 mM L-cysteine and IscS (20 μM) were added in place of Li_2_S.

### Quaternary structure determination

The quaternary structure of Mrp_ORP__CT was determined using a Superdex 200 10/300 GL size exclusion column (GE-Healthcare). The mobile phase used was 100 mM Tris-HCl, pH 7.5 and 500 mM NaCl and protein was injected on the colum at a flow-rate.of 1 mL/min. The standard used to create a standard curve were β-amylase (200 kDa) albumin (66 kDa) and carbonic anhydrase (29 kDa).

### [Fe-S] Cluster Transfer from Mrp_ORP_ to Orp3-Orp4-Orp8 Proteins Complex

Apo-Orp3-Orp4-Orp8 was obtained after 1 h incubation in a 50-fold molar excess of Na_2_S_2_O_4_ and EDTA. The resulting protein solution was loaded onto a Hitrap Desalting column (GE Healthcare) and eluted in 0.1 mM Tris-HCl pH 8, 50 mM KCl, 3 mM DTT. Apo-Orp3-Orp4-Orp8 was mixed in the anaerobic chamber for 1 h with Holo-Dde3202 in buffer C (0.1 mM Tris-HCl pH 7.5, 500 mM NaCl, 5 mM DTT) in a high molar ratio to give sufficient amounts of iron and sulfur per Apo-Orp3 monomer. The solution containing each protein was loaded onto a 1 mL Ni-Sepharose affinity column (His-Trap HP, GE Healthcare). Dde3202, which does not contain a poly-Histidine tag, was recovered in the flow-through, while Orp3-His-tagged was eluted with buffer C containing 100 mM of imidazole. The eluted fraction containing Orp3 with co-eluted Orp4 and Orp8 was analyzed and an UV-visible spectrum was recorded.

### Aconitase Activity

Aconitase activity assays were performed in the Coy anaerobic chamber, by mixing 0 to 40 μM of Holo-Mrp_ORP_ and 2 μM of Apo-AcnB. 50 mM Tris-HCl pH 7.5, 1 mM MnCl_2_, 25 mM citrate, 0.25 mM NADP^+^, 1.7 U of isocitrate dehydrogenase were added to proteins in 1 mL final volume in a quartz cuvette closed with a rubber cap. AcnB activity was assayed by following the formation of NADPH in the coupled assay as an increase in absorbance at 340 nm^29^.

### Phylogenetic Analysis

According to the INTERPRO database (v62, 17 March 2017), the Mrp_ORP_ (DVU2109) is composed of two domains: a P-loop containing nucleoside triphosphate hydrolase domain (IPR027417) and a dinitrogenase iron-molybdenum cofactor biosynthesis domain (IPR003731). DVU2109 belongs, together with DVU1847 and DVU2330, to the Mrp/NBP35 ATP-binding protein family (IPR019591), which contained 17300 sequences from known cellular organisms and 211 unclassified sequences. The 17300 sequences associated to cellular organisms were retrieved and aligned with MAFFT v7.309 (default parameters)^48^. The quality of the resulting alignment was verified with SEAVIEW v4.6.1^49^. 1,146 partial or poor-quality sequences were discarded. The 16,154 conserved sequences, among which 99 combine IPR027417 and the IPR003731, were realigned with MAFFT (default parameters). The resulting alignment was trimmed with BMGE (BLOSUM30 option)^50^ and used to infer the phylogeny of the Mrp/Nbp35 ATP-binding protein family with FASTTREE v2.1.9^51^. Among the 17,300 protein sequences from known cellular organisms retrieved from INTERPRO, the 110 sequences displaying the highest similarity with Mrp_orp_ were identified with BLASTP (default parameters)^52^. The corresponding sequences were aligned with MAFFT using the accurate option L-INS-I option, which allowed accurate multiple alignment construction. The resulting alignment was trimmed with BMGE (BLOSUM30 option) and used to infer a maximum likelihood tree with IQ-TREE v1.5.3^53^ using the LG + I + G4 evolutionary model as suggested by the model selection tool implemented in IQ-TREE (BIC criterion). The robustness of the resulting tree was assessed with the non-parametric bootstrap procedure implemented in IQ-TREE (100 replicates of the original dataset)^54^.

### Microscopy Experiments

The 3 fusion GFP strains were grown until the middle of the exponential growth phase (OD_600nm_ of approximately 0.4 to 0.5) in medium C. Cells were concentrated 2 times by centrifugation. The buffer used for this concentration contained 10 mM Tris-HCl (pH 7.6), 8 mM MgSO_4_, 1 mM KH_2_PO_4_. In order to stain DNA, this buffer was supplemented with 5 ng/μL of 4’,6-diamidino-2-phenylindole (DAPI). After 20 min of incubation in the dark, the cells were washed three times in TPM buffer. The DNA was stained under anaerobic conditions to limit the exposure of the cells to air. The pictures were acquired after 10 min of air exposure, which was required for oxygen GFP maturation. The cells were placed between a coverslip and an agar pad of 2% agarose. Pictures were acquired with a Nikon TiE-PFS inverted epifluorescence microscope, 100x NA1.3 oil PhC objective (Nikon), and Hamamatsu Orca-R2 camera. For fluorescent images, a Nikon intenselight C-HGFI fluorescence lamp was used. Specific filters were used for each wavelength (Semrok HQ DAPI/CFP/GFP/YFP/TxRed). Image processing was controlled by the NIS-Element software (Nikon).

### EPR Spectroscopy

The EPR spectra of 0.1 mM Mrp_ORP__CT, in 100 mM Tris-HCl, 500 mM NaCl, 3 mM DTT pH 8.1, were recorded on a X-band Bruker EMX spectrometer equipped with a rectangular cavity (model ER 4102 T) and an Oxford Instruments continuous liquid helium flow cryostat. The EPR sample was reduced with a solution of sodium dithionite prepared in 100 mM Tris-HCl pH 7.6. The experimental conditions used for spectral acquisition are described in the Figure legend. The EPR samples were prepared under anoxic conditions either inside a COY or a Mbraun anaerobic chamber.

### Electrochemistry Electrode Preparation

Before each experiment, the pyrolytic graphite edge electrode (PGE) surface was polished with 1 and 0.5 µm alumina slurry, sonicated for 5 minutes and rinsed with deionized water. Then, the surface of the PGE was coated with 1.5 µL polymyxin B sulphate (2 mM) and a 3 µL drop of Mrp_ORP__CT (121 µM) were deposited and left to dry for 45 min before electrode immersion in the electrolyte solution.

### Electrochemical Measurements

All the electrochemical experiments were conducted inside an anaerobic chamber (MBraun) at room temperature, with 100 mM Tris-HCl 8.1, 500 mM NaCl, 2.5 mM desthiobiotin, 3 mM DTT used as electrolyte properly flushed with argon before entering in the chamber. A three-electrode configuration system, containing a reference electrode, Ag/AgCl (+205 mV *vs* SHE), a secondary electrode, platinum wire, and a work electrode, PGE, were used. To measure the cyclic voltammograms a µautolab (ECO Chemie, Utrecht, The Netherlands) was used being the data collect and analyzed on GPES software package (ECO chemie). Cyclic voltammetric measurements were performed on a potential window from +0.1 to −0.9 V (*vs* SHE), and the scan rate dependence investigated between 0.005 and 0.1 V s^−1^.

### Protein and Metal Quantifications for Mrp_ORP__CT

Iron quantification was performed by inductively coupled plasma (ICP) emission analysis in a Jobin-Yvon (Ultima) instrument (at UCIBIO), using the Reagecom 23 ICP multielements as standard solution in a concentration range of 0.05 to 3 ppm. Total Protein was quantified using the Pierce^TM^ 660 nm Protein Assay (Thermo), with bovine serum albumin (Sigma) as standard protein.

## Supplementary information

Supplemental Information

Table S1

Table S2

Table S3
