# Supplementary material for: The bacterial MrpORP is a novel Mrp/NBP35 protein involved in iron-sulfur biogenesis
Source: Sci Rep. 2019 Jan 24;9:712. doi: 10.1038/s41598-018-37021-8 (PMC6345978; doi:10.1038/s41598-018-37021-8)
Supplement: Supplementary file 1 — Supplemental Information [file 41598_2018_37021_MOESM1_ESM.docx]

**Supplemental Material**

**The bacterial Mrp_ORP_ is a novel Mrp/NBP35 protein involved in iron-sulfur biogenesis**

**Romain Pardoux^1^, Anouchka Fievet^1^, Cíntia Carreira^2^, Céline Brochier-Armanet^3^, Odile Valette^1^, Zorah Dermoun^1^, Béatrice Py^1^, Alain Dolla^1^, Sofia R. Pauleta^2^, Corinne Aubert^1★^**

*** Correspondence:** Corresponding Author: aubert@imm.cnrs.fr

Supplemental Figures and Tables:

This document :

Supplemental tables : S4 and S5

Supplemental figures : 1-6.

Additional files :

Table S1.xls : Taxonomic distribution of proteins belonging to the Mrp/NBP35 ATP-binding protein family (IPR019591).

Table S2.xls : Domain architecture displayed by the members of the Mrp/NBP35 ATP-binding protein family (IPR019591).

Table S3.xls : Taxonomic distribution of protein sequences combining IPR027417 and IPR003731 (or related) domains.

**SUPPLEMENTAL TABLES AND TABLES LEGENDS**

**Table S4.** Bacterial strains and plasmids used in this study.

| ***Strains or***  ***plasmids*** | ***Characteristics*** | ***Reference***  ***or source*** |
| --- | --- | --- |
| **Strains**  *E. coli* TG1  *Desulfovibrio vulgaris* Hildenborough (DvH)  *Desulfovibrio desulfuricans* G20  DVH (ORP_3_-strep)  DVH (Cter-Mrp_ORP_-strep)  **Plasmids**  pJF119  pBMC6  pBMC6C3::3202strep  pBMC6C3::3202 C215A/C218Astrep  pBMC6C3::Cter 2109strep  pBMC6C3::2103His  pjf1119-3202His  pjf1119-Nter3202His  pjf1119-2109His  pjf119-Mrp  pNot19Cm-Mob-XS-*mrp_orp_-gfp*  pNot19Cm-Mob-XS *ploop-ntpase-gfp*  pNot19Cm-Mob-XS *di-nase-gfp* | K12, Δ(*lac-pro*), *supE*, *thi*, *hsdD5* (F’, *traD36*, *proA^+^B^+^*, *lacI^Q^*, *lacZΔM15*)  *pir]*  Wild-type strain  Wild-type strain  Contains the *DVU2103* gene fused to a C-terminal Strep-TagII tag in DVH (DVH::p2103-strep)    Contains the *DVU2109* gene from residue 351 to residue 487 fused to a C-terminal Strep-TagII tag in DVH  (DVH::p2109-strep)  Cloning vector, RBS, P_tac_, Amp^R^  Broad-host-range vector, Km^R^  Contains the *pcyc-DDE3202* gene fusion on a 1500pb *EcoR*I-*Hind*III insert gene fused to an C-terminal streptagII-tag in pBMC6  Contains the *pcyc-DDE3202* mutated gene fusion on a 1500pb *EcoR*I-*Hind*III insert gene fused to an C-terminal streptagII-tag in pBMC6  Contains the *cyc-dvu2109cter* gene fusion on a 1000pb *EcoR*I-*Hind*III insert gene fused to an C-terminal streptagII-tag in pBMC6  Contains the *pcyc-dvu2105-dvu2104-dvu2103* gene fusion on a 2500pb *EcoR*I-*Hind*III insert gene fused to an C-terminal His-tag in pBMC6  Contains the *dde3202* gene fusion on a 1250pb *EcoR*I-*BamH*I insert gene fused to an C-terminal His-tag  Contains the *dde3202Nter* gene fusion on a 800pb *EcoR*I-*BamH*I insert gene fused to an C-terminal His-tag  Contains the *dvu2109* gene fusion on a 1350pb *EcoR*I-*BamH*I insert gene fused to an C-terminal His-tag  Contains the *b2113mrp* gene fusion on a 1110pb *EcoR*I-*BamH*I insert gene fused to an C-terminal His-tag  Contains the *mrp_orp_-gfp* gene fusion on a 2100pb *Xho*I-*Nde*I insert gene.  Contains the *ploop-ntpase-gfp* gene fusion on a 1800pb *Xho*I-*Spe*I insert gene.  Contains the *di-nase-gfp* gene fusion on a 300pb *Xho*I-*Spe*I insert gene. | Sambrook and Russell (2001)  Postgate (1984)  Postgate (1984)  This Study  This Study  Albermann et al, 2010  Rousset et al, 1998  This Study  This Study  This Study  This Study  This Study  This Study  This Study  This Study  This Study  This study |

Postgate, J.R (1984) in The Sulphate-reducing bacteria, 2^nd^ ed; pp12-13, Cambridge University Press, Cambridge, UK.

Woodcock, D.M., Crowther, D.M., Doherty, J., Jefferson,S., DeCruz, E., Noyer-Weidner, M., Smith, S.S., Michael, M.Z., and Graham, M.W., Nucl. Acids Res. (1989) 17, 3469-3478

Rousset, M , [Casalot L](http://www.ncbi.nlm.nih.gov/pubmed/?term=Casalot%20L%5BAuthor%5D&cauthor=true&cauthor_uid=9514705), [Rapp-Giles BJ](http://www.ncbi.nlm.nih.gov/pubmed/?term=Rapp-Giles%20BJ%5BAuthor%5D&cauthor=true&cauthor_uid=9514705), [Dermoun Z](http://www.ncbi.nlm.nih.gov/pubmed/?term=Dermoun%20Z%5BAuthor%5D&cauthor=true&cauthor_uid=9514705), [de Philip P](http://www.ncbi.nlm.nih.gov/pubmed/?term=de%20Philip%20P%5BAuthor%5D&cauthor=true&cauthor_uid=9514705), [Bélaich JP](http://www.ncbi.nlm.nih.gov/pubmed/?term=B%C3%A9laich%20JP%5BAuthor%5D&cauthor=true&cauthor_uid=9514705), [Wall JD](http://www.ncbi.nlm.nih.gov/pubmed/?term=Wall%20JD%5BAuthor%5D&cauthor=true&cauthor_uid=9514705).[Plasmid.](http://www.ncbi.nlm.nih.gov/pubmed/9514705) 1998; 39(2):114-22.

Albermann C, Trachtmann N, Sprenger G (2010) Biotechnol J 5:32-38.

Sambrook J and Russell DW (2001) The Quarterly Review of Biology. 76(3):348-349.

**Table S5.** Primers used in this study.

| Primer | Sequence (5’-3’) |
| --- | --- |
| **For overproduction in *E.coli***  2109(1)*EcoR*I  StrepCter2109_*BamH*I  Mrp_*Ecor*I  Mrp_*BamH*I  3202R_(302)8His*BamH*1  3202F_EcoRI  3202R_8His*BamH*I  **For site-directed mutagenesis**  3202CysmutF  3202cysmutR  **For production in DvH**  2103-C3_dir  2103-8His-*Sac*I_comp  3202F_*Nde*I  3202strepR_*Sac*I  Cter2109_*Sac*I  Strep2109del*Nde*I  2109(351)*Nde*I  Cter2109(351)strep  **For microscopy experiments**  NterDVU2109_*Xho*I  CterDVU2109_*Nde*I  domCter2109-dir-*Xho*I  CterGFP-*Spe*I  Nter2109-XhoI | 5’ATGCATGAATTCATGAGTACCGTGAACGGCGCGAAC-3’  5’-GGATGAGCTCTCAATGGTCGCAGGTGTTGGCAC-3’  5’-ATGCATGAATTCATGAACGAACAATCCCAGGCCAAA-3’  5’-TAATCGGATCCTTAGACCGCGCGGAAGGAAATCTCG-3’  5’-TAATCGGATCCTTAGTGGTGGTGGTGGTGGTGGTGGTGGCCTTCGGCCTGCGGTGCCGGCAC-3’  5’-ATGCATGAATTCATGAGTGAATCTTGCGGTTGCTCC_3’  5’-TAATCGGATCCTTAGTGGTGGTGGTGGTGGTGGTGGTGGTGATGGTCGCAGGTGTTTGCTCCGGC-3’  5’-CAGGCTGACCATGTGGAACCGCTGTT-3’  5’-CGGAGCGGCAAAGCCGCTCATATTTTCG-3’  5’-CTTTGTGAAGGAGGTAGTTCGATCATGCCATACGGAGATGGAAC-3’  5’GCATGAGCTCTCAGTGGTGGTGGTGGTGGTGGTGGTGTGCTGCGACGAGTGCCAGCAACTGGTTCCAAACTCCGTTGAACCGTTGCC-3’  5’-CATATGCATCATATGAGTGAATCTTGCGGTTGCTCC-3’  5’-TAATCGAGCTCTTATTTTTCGAACTGCGGGTGGCTCCAATGGTCGCAGGTGTTTGCTCCGGC-3’  5’-GGATGAGCTCTCAATGGTCGCAGGTGTTGGCAC-3’  5’TAATCGAGCTCTTATTTTTCGAACTGCGGGTGGCTCCAAGCGCTATGGTCGCAGGTGTTGGCAAGGGTGAGGGTTCCGGCCATGTGTGCTGCG-3’  5’-ATGCTACATATGCCCACGGCCCCCACCCGC-3’  5’-TAATCGAGCTCTTATTTTTCGGGGTGGCTCCAAGCGCTGGTCGCGGCGGTCTTGCCCGTTCCG-3’  5’-ATGCATGCCTCGACATGAGTACCGTGAACGGCGC-3’  5’-ATGCATGCCATATGATGGTCGCAGGTGTTGGCACCAAGG-3’  5’-GATCCTCGAGATGCCCACGGCCCCCACCCGCAACGAATCA-3’  5’-CGATACTAGTTTACTTGTACAGCTCGTCCAT-3’  5’-ATGCATGCCTCGAGATGAGTACCGTGAACGGCGCG -3’ |

**Figure S1**: **Unrooted Maximum likelihood tree of the 110 sequences displaying highest similarity with Orp9** (110 sequences, 233 amino acid positions). Functional domain organization in sequences is displayed. The scale bar indicates the average number of substitutions per site. Circles associated to branches are proportional to bootstrap values. For clarity bootstrap values lower than 75% were omitted.

**Figure S2 : Genomic *orp* gene cluster organization in *Desulfovibrio vulgaris* Hildenborough and *Desulfovibrio desulfuricans*** G20. The arrows represent the transcriptional units (Fiévet et al, 2011 ; Carepo et al, 2015)

**
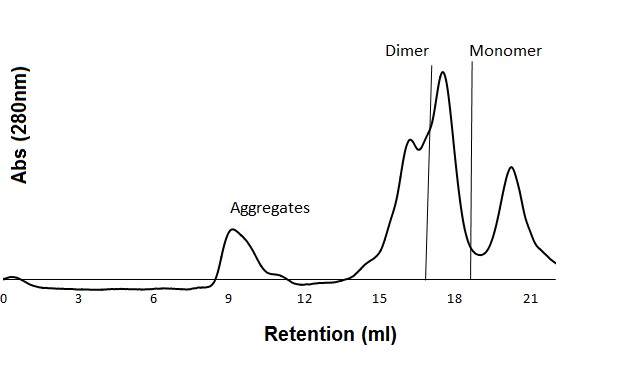
**

**Figure S3 :The quaternary structure of the Mrp_ORP__CT determined by gel filtration**. Retention volumes expected for monomers and dimers of Mrp_ORP__CT have been added on the figure. The gel filtration conditions are as follows: solide phase, Superdex 200; mobile phase, 100mM Tris-HCl ph7,5, 500mM NaCl; flow-rate 1mL/min.


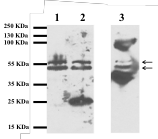


**Figure S4 : Western blot of Mrp_ORP_-GFP using antibodies against GFP protein**. DvH wild-type (lane 1), DvH pC3::*egfp* (lane 2) and DvH Mrp_ORP_-GFP (lane 3). Arrows represent non-specific bands.

ThenaDRAFT_0395 --------------------------------------------MADKD---------KN 7

DVU2109_DVH MSTVNGANIQSEEQHTGGCSAGCSGGSTGGSTDGSTGGNTQQGCSCDTHDEQRDGSVAAQ 60

Dde_3202 -------------MS-ESCG--CSAGGNC----------SSGGCH--ENKSPE------- 25

Desal_0620 -------------MSDHACGSCSSSGSGC----------SSSGCS--EGCSPE------- 28

Dret_0440 -------------MAEHGCSSCSSGGSGC----------QGGK------NEQE------- 24

ThenaDRAFT_0395 KEKLERLENFLKKVSNKIMVMSGKGGVGKSTVAANLAVFLSNRGYKVGLLDVDVHGPSIG 67

DVU2109_DVH TFGEEGPVKTLGRIGSKLVVLSGKGGVGKSTVAVNLAVGLARAGRKVGLLDVDVHGPSVP 120

Dde_3202 ---DLRLESSVSRIRNKVVVMSGKGGVGKSTIAANIAVSLALAGQKVGLLDVDVHGPSIP 82

Desal_0620 ---DMKLKKALSRIKHKIVVISGKGGVGKSTVATNIAVALSLAGKQVGLLDVDVHGPSVP 55

Dret_0440 ---DQKLQNCLSRIKNKFVVLSGKGGVGKSTVAVNLAASLAMAGQKVGLLDVDVHGPSVP 51

. : :: *.:*:**********:*.*:*. *: * :************:

ThenaDRAFT_0395 TIMGIVWQRIYPSGEMLKPVLWSKNLKVVSVQFLLENPDDAIIWRGPIKIGIINQFLSDV 127

DVU2109_DVH RLLGLTGTRPMIGEDAMYPVGWRNNLRVMSLGFFLPDPEQAVIWRGPVKMGLIRHFLTEV 180

Dde_3202 RLLGLDKAEIRMEERSLLPVPWNANLSVMSVGFMIPDPQQAVIWRGPVKMGFIKQMLSEV 142

Desal_0620 RLLSLQDEKPHIGHEVIEPISWSSNLWVMSLGFMLPSKDDPVIWRGPVKIGMIKQFVQDV 115

Dret_0440 RLLSLGQSKPHLDNQCIEPIQWDKNLWVMSLGFMLPNANEAVIWRGPVKMGLIKQFLEDV 111

::.: . : *: * ** *:*: *:: . :: :*****:*:*:*.::: :*

ThenaDRAFT_0395 DWGELDYLIIDSPPGTGDEPLTIAQTI-PDCKALIVTTPQKLSLADVRKSLTFCKQVNID 186

DVU2109_DVH RWGDLDHLVVDCPPGTGDEPLSVLQLLGTDAQAVIVTTPQGVAVDDVRRSVGFCRELGNP 240

Dde_3202 AWGDLDFLVVDCPPGTGDEPLSVLQLLGTDARAVIVTTPQAVAVDDVRRSIGFCRELGNP 202

Desal_0620 AWNDLDFLVVDCPPGTGDEPLSALQTLGQDAHAVIVTTPQGVAIDDVRRSVNFCKQVGNP 175

Dret_0440 AWGDLDYLIVDCPPGTGDEPLSTLQLLGQDAEAVIVTTPQGVAVDDVRRSVTFCQQLGNP 171

*.:**.*::*.*********: * : *..*:****** ::: ***:*: **:::.

ThenaDRAFT_0395 VLGVIENMSGFV**CP**N**C**GTVHNIFKSGGGDELSKQYKIDFLGKIPIDPKIVEESDEGNLLD 246

DVU2109_DVH ILGIVENMGGYV**CP**K**C**GELTPLFPAGGGEALAAEQGVTFLGRIPLHPDLTSAGDAGRSLY 300

Dde_3202 IAGVVENMSGFA**CP**Q**C**DHVEPLFGQGGGEALAKETNVPFLGAVPATSLMSRCGDKGLVFV 262

Desal_0620 VLGIVENMSGFV**CP**D**C**GNVHDIFNSGGGEELAKETGVKFLGRVPLDPEVGRSGDEGYPII 235

Dret_0440 VFGIVENMSGFV**CP**S**C**KETVDIFTSGGGEELASEMHARFLGRIPLDPEIVRAGDEGYVFV 231

: *::***.*:.**.* :* ***: *: : *** :* : .* * :

ThenaDRAFT_0395 KYNGK--VKEIMNEIVDKIINKL------------------------------------- 267

DVU2109_DVH EADAAHPIVRALAPIVERAAATLHASDIRHESTTGPQSEAAPGTGKTAATMPTAPTRNES 360

Dde_3202 QAQPENPVAEAIGRIVKPLLAHA---GTLHEREG-------------------------- 293

Desal_0620 RTDHESPTGKALNTIIKPMLNLT---ETLQENNEM------------------------- 267

Dret_0440 KTHHESPAAQAVGSIVKPMLAKQ---ELLQEKPAG------------------------- 263

. . . : *:.

ThenaDRAFT_0395 -----SKNNKGGESFMRIAIPMAQGEL**C**A**HFGHCE**VFGIADVE--DGKIVKEEYL**TPPPH 320**

DVU2109_DVH CAPATNHPDTNGDTTMKIAIPVAGDVL**C**Q**HFGH**C**E**RFALIDVDPATGSVTGRNDV**TPPPH 420**

Dde_3202 -----AVPAPQAEGTVKVALPLANGSL**C**Q**HFGHCE**QFAIVTADTTAKTVLGTENI**TPPPH 348**

Desal_0620 ---PKPDELQAKNGMIRIAVPVAAGKL**C**M**HFGHCE**QFALMDIDVATKGIVATNME**TPPPH 324**

Dret_0440 ---QT-APAPEADGEMLIAVPVAQGQL**C**M**HFGHCE**QFALVRVDTANKTVLGTEMH**TPPPH 319**

: : :*:*:* . ** ****** *.: : : : *****

ThenaDRAFT_0395 A**PG**VIPNWLAKQKVNVVLTGGMGPMAKNLMRQNGIEVITGVSGGTLRDVVEAYLNNKLVI 380

DVU2109_DVH E**PG**LLPVWLSEKGANLVIAGGMGARARALLEERGVKVLIGAPAAAPEAIVAAHMAGTLTL 480

Dde_3202 E**PG**VLPRFLAEQGVNVVLAGGMGARAQSLFTGQGINVVTGISGGSPHEVVSAWMQGSLTA 408

Desal_0620 E**PG**VLPKWIADQGVQLVLAGGMGSRAQSLFTDAGVKVIVGSPAEAPENVVSSYLAGTLQT 384

Dret_0440 E**PG**VLPKWLADQQANLVIAGGMGSRAQSLFTQHGVKVITGAQPGAAEEVVGSYLAGTLQT 379

**::* :::.: .::*::**** *: *: *::*: * : . :* : : ..*

ThenaDRAFT_0395 GENSCDHEHGKGHAHKH 397

DVU2109_DVH GANTCDH---------- 487

Dde_3202 GANTCDH---------- 415

Desal_0620 GSNTCDH---------- 391

Dret_0440 GQNICDH---------- 386

* * ***

**Figure S5**: **Protein sequences alignment of C-terminal domains of two-domain Mrp/AbpC-like homologs with full length IPR003731 proteins.** Bacterial sequences are from Dde (DDE_3202; Refseq accession no. _[YP_389691.1](http://www.ncbi.nlm.nih.gov/entrez/viewer.fcgi?db=protein&val=YP_389691.1)), DvH (DVU2109 ; Refseq accession no. [YP_011322.1](http://www.ncbi.nlm.nih.gov/entrez/viewer.fcgi?db=protein&val=YP_011322.1)), *Desulfohalobium retbaense* DSM 5692 (Dret_0440 ; Refseq accession no. [YP_003197315.1](http://www.ncbi.nlm.nih.gov/entrez/viewer.fcgi?db=protein&val=YP_003197315.1)), [*Desulfovibrio salexigens DSM 2638*](http://www.microbesonline.org/cgi-bin/genomeInfo.cgi?taxId=526222) (Desal_0620 ; Refseq accession no. [YP_002990225.1](http://www.ncbi.nlm.nih.gov/entrez/viewer.fcgi?db=protein&val=YP_002990225.1)) and *Thermodesulfobium narugense* Na82, DSM 14796 (ThenaDRAFT_0395, Refseq accession no. VIMSS ID 11113744).


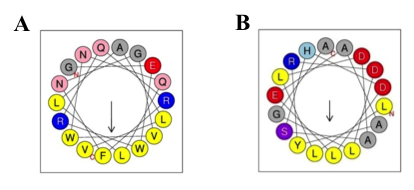


**Figure S6**: Helical-wheel representation of the putative Orp3 (A) (higher probability) and Orp4 (B) (lower probability) membrane targeting sequence in yellow (HeliQuest Software, Gautier R., Douguet D., Antonny B. and Drin G. HELIQUEST: a web server to screen sequences with specific α-helical properties. Bioinformatics. 2008 Sep 15;24(18):2101-2.
